# Direct Visualization of Single Nuclear Pore Complex Proteins Using Genetically‐Encoded Probes for DNA‐PAINT

**DOI:** 10.1002/anie.201905685

**Published:** 2019-08-21

**Authors:** Thomas Schlichthaerle, Maximilian T. Strauss, Florian Schueder, Alexander Auer, Bianca Nijmeijer, Moritz Kueblbeck, Vilma Jimenez Sabinina, Jervis V. Thevathasan, Jonas Ries, Jan Ellenberg, Ralf Jungmann

**Affiliations:** ^1^ Faculty of Physics and Center for Nanoscience LMU Munich Geschwister-Scholl-Platz 1 80539 Munich Germany; ^2^ Max Planck Institute of Biochemistry Am Klopferspitz 18 82152 Martinsried Germany; ^3^ Cell Biology and Biophysics Unit European Molecular Biology Laboratory (EMBL) Meyerhofstraße 1 69117 Heidelberg Germany

**Keywords:** DNA-PAINT, genetically encoded tags, nuclear pore complex, single-molecule imaging, super-resolution microscopy

## Abstract

The nuclear pore complex (NPC) is one of the largest and most complex protein assemblies in the cell and, among other functions, serves as the gatekeeper of nucleocytoplasmic transport. Unraveling its molecular architecture and functioning has been an active research topic for decades with recent cryogenic electron microscopy and super‐resolution studies advancing our understanding of the architecture of the NPC complex. However, the specific and direct visualization of single copies of NPC proteins is thus far elusive. Herein, we combine genetically‐encoded self‐labeling enzymes such as SNAP‐tag and HaloTag with DNA‐PAINT microscopy. We resolve single copies of nucleoporins in the human Y‐complex in three dimensions with a precision of circa 3 nm, enabling studies of multicomponent complexes on the level of single proteins in cells using optical fluorescence microscopy.

Super‐resolution techniques allow diffraction‐unlimited fluorescence imaging^1^ and with recent advancements, true biomolecular resolution is well within reach.^2^ One implementation of single‐molecule localization microscopy (SMLM) is called DNA points accumulation in nanoscale topography2b (DNA‐PAINT), where dye‐labeled DNA strands (called “imager” strands) transiently bind to their complements (called “docking” strands) on a target of interest, thus creating the typical “blinking” used in SMLM to achieve super‐resolution. While localization precisions down to approximately one nanometer (basically the size of a single dye molecule) are now routinely achievable from a technology perspective, this respectable spatial resolution has yet to be translated to cell biological research. Currently, this is mainly hampered by the lack of small and efficient protein labels. Recent developments of nanobody‐ or aptamer‐based tagging approaches^3^ are providing an attractive route ahead, however both approaches are not yet deploying their full potential either due to limited binder availability (in the case of nanobodies) or lack of large‐scale analysis of suitable super‐resolution probes (in the aptamer case).

While we are convinced that some of these issues might be resolved in the future, we introduce herein the combination of widely‐used, genetically‐encoded self‐labeling enzymes such as SNAP‐tag^4^ and HaloTag^5^ with DNA‐PAINT to enable 1:1 labeling of single proteins in the nuclear pore complex (NPC) using ligand‐conjugated DNA‐PAINT docking strands. The NPC is responsible for the control of nucleocytoplasmic transport and a highly complex and sophisticated protein assembly. NPCs contain multiple copies of approximately 30 different nuclear pore proteins called nucleoporins (NUPs) and have an estimated total molecular mass of about 120 MDa, placing NPCs among the largest cellular protein complexes.^6^ Owing to their diverse function in controlling molecular transport between the nucleus and the cytoplasm, NPCs are a major target for structural biology research with characterization by for example, cryogenic electron microscopy^6^ (cryo‐EM) or optical super‐resolution techniques.^7^ State‐of‐the‐art cryo‐EM studies,^8^ reaching impressive pseudo‐atomic resolution, have advanced our structural understanding in recent years. It is now possible to not only elucidate how NUPs in NPCs are arranged, but also to shed light on how structural changes of NPCs are connected to their dysfunction.^9^ However, even with recent advancements in cryo‐EM instrumentation, molecular specificity necessary to resolve single NUPs in NPCs proves still elusive, mainly due to the lack of high protein‐specific contrast. Fluorescence‐based techniques on the other hand offer exquisite molecular contrast and specificity owing to the use of dye‐labeled affinity reagents targeting single protein copies in cells. However, until recently, the necessary resolution to spatially resolve single small proteins in a larger complex has not been achieved because of limitations in labeling (small and efficient probes) and imaging technology (providing sub‐10‐nm spatial resolution). In order to spatially resolve sub‐10‐nm distances using SMLM, one needs to obtain a localization precision of circa 4 nm. This is readily achievable with DNA‐PAINT, as a comparably large number of photons is available for localization per single binding—that is, blinking—event. While one can easily reach tens of thousands of photons per blinking event with DNA‐PAINT, this is hard to achieve using STORM. Furthermore, the intrinsic resistance of DNA‐PAINT to photobleaching enables repetitive localizations with high precision, while in the STORM case, the available photon budget is limited by a few fixed, target‐bound fluorophores. Herein, we thus combine DNA‐PAINT microscopy with small, genetically‐encoded self‐labeling enzymes such as SNAP‐ and HaloTag to overcome limitations in optical super‐resolution microscopy.

We present a straightforward protocol to target these tags in a variety of engineered cell lines using the DNA‐conjugated ligands benzylguanine (BG) and chloroalkane against SNAP‐tag^4^ and HaloTag,^5^ respectively (Figure 1 a and b). We investigate the achievable labeling precision and reduction of linkage error of SNAP‐tag and HaloTag, examine their performance in contrast to DNA‐conjugated nanobodies against GFP‐tagged proteins in single NPCs and further compare them to primary and secondary antibody labeling. Finally, we resolve, for the first time, single copies of NUP96 proteins in the Y‐complex of the NPC, spaced only circa 12 nm apart.

**Figure 1:**
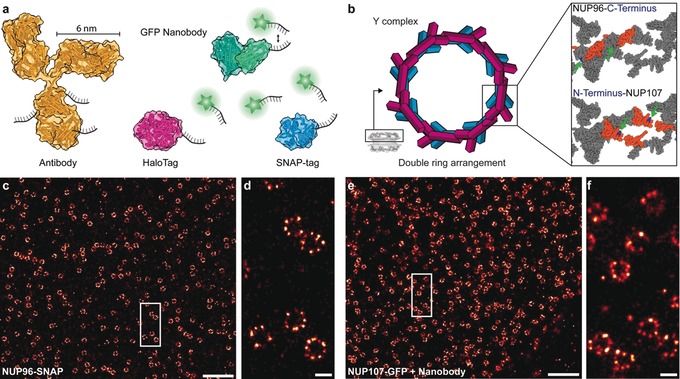
a) Comparison of different labeling probes (secondary antibody: yellow, GFP nanobody: green, HaloTag: magenta, SNAP‐tag: blue) conjugated with DNA strands for DNA‐PAINT imaging (cartoons are based on protein database (PDB) entries: Secondary antibody (1IGT), GFP nanobody (3K1K), HaloTag (4KAF), SNAP‐tag (3KZZ)). Proteins are to scale. b) NPCs contain 16 copies of NUP96 and NUP107 in the cytoplasmic as well as the nuclear ring. Top right: C‐terminally‐labeled (blue, highlighted by green arrows) NUP96 structure (orange) highlighted in the zoom‐in of a symmetry center on the ring (ca. 12 nm apart). Bottom right: N‐terminally‐labeled (blue, highlighted by green arrows), NUP107 structure (orange) in the zoom‐in of a symmetry center on the ring (ca. 12 nm apart). Distances and cartoons derived from PDB entry: Nup(5A9Q). c) DNA‐PAINT overview image of NUP96‐SNAP in U2OS cells. d) Zoom‐in of highlighted area reveals the arrangement of NUP96 in NPCs. e) DNA‐PAINT overview image of NUP107‐GFP in HeLa cells. f) Zoom‐in of highlighted area. Scale bars: 5 μm (c, e), 100 nm (d, f).

To implement genetically‐encoded self‐labeling tags for DNA‐PAINT, we first assayed our ability to use BG‐modified docking strands to target SNAP‐tags C‐terminally fused to NUP96 proteins in U2OS cell lines created by CRISPR/Cas9 engineering.^10^ Labeling was performed post‐fixation and ‐permeabilization using standard labeling protocols7b adapted for DNA‐PAINT (see Online Methods). The resulting 2D DNA‐PAINT image is shown in Figure 1 c. A zoom‐in reveals the expected 8‐fold symmetry of NUP96 proteins in the super‐resolution micrograph (Figure 1 d). We then performed labeling of NUP107‐GFP fusion proteins using a DNA‐conjugated anti‐GFP nanobody^11^ and obtained qualitatively similar results (Figure 1 e–f, see also Supplementary Figure 1 in the Supporting Information for zoom‐outs and comparison to diffraction‐limited data).

To evaluate labeling quality and precision in a quantitative manner, we next compared results of more traditional labeling of NUP107‐GFP using primary‐secondary antibodies to those of NUP96‐SNAP, NUP96‐Halo, NUP107‐SNAP, and NUP107‐GFP cell lines targeted with their respective small ligands. The NPC architecture presents a well‐suited model to benchmark novel labeling approaches with regards to overall labeling efficiency and limits of spatial resolution, in a sense similar to an in vitro DNA origami calibration standard,^12^ but inside a cell. Previous EM studies revealed that NUP96 and NUP107 proteins are present in the Y‐complex, which forms the cytoplasmic as well as nuclear NPC double ring arrangement in an 8‐fold symmetry. The two double rings are spaced approximately 50 nm apart with each side containing 16 protein copies.8a, 13 The two copies of the proteins in each symmetry center are arranged in Y‐complexes spaced circa 12 nm apart (Figure 1 b). In order to quantitatively compare different labeling approaches, we first acquired 2D DNA‐PAINT data using identical image acquisition parameters (see Supplementary Tables 1–3 in the Supporting Information for details). Next, we selected single NPC structures in the reconstructed super‐resolution image, aligned them on top of each other (that is, the center of the NPC rings, thus creating a sum image) and performed a radial distance measurement over all localizations. This analysis yields two observables for comparison; first, the median fitted ring radius and second, the width of this distribution. Dissimilar fitted radii for the same protein labeled using different tags are a measure for potential systematic biases introduced by a preferential orientation of the labeling probes. The width of the distribution on the other hand is a proxy for label‐size‐induced linkage error, that is, broader distributions originate from “larger” labeling probes. Our data in Figure 2 provides a quantitative comparison of NUP107‐SNAP, NUP107‐GFP, NUP96‐SNAP, and NUP96‐Halo cell lines targeted with their respective DNA‐conjugated labeling probes (see also Supplementary Figures 2–6 in the Supporting Information). Furthermore, we compare our results with NUP107‐GFP labeled using primary and DNA‐conjugated secondary antibodies. We obtained radii of 53.7±13.1 nm for NUP107‐SNAP (Figure 2 a and Supplementary Figure 2 in the Supporting Information) and 54.6±11.9 nm for NUP107‐GFP (nanobody staining) (Figure 2 b and Supplementary Figure 3 in the Supporting Information), as well as 55.9±12.6 nm for NUP96‐SNAP (Figure 2 c and Supplementary Figure 4 in the Supporting Information) and 56.2±10.2 nm for NUP96‐Halo (Figure 2 d and Supplementary Figure 5 in the Supporting Information), in close overall agreement to earlier EM‐ and fluorescence‐based studies.7b, 8a For the antibody‐stained sample against NUP107‐GFP (Figure 2 e and Supplementary Figure 6 in the Supporting Information), we obtained a considerably larger radius of 65.9 nm. This could be explained by primary and DNA‐conjugated secondary antibodies potentially binding preferentially towards the outside of the NPCs. However, not only did the antibody‐stained samples yield a larger apparent NPC radius, also the measured width of the distribution (18 nm) was larger compared to the genetically‐encoded tags due to the increased size of the antibodies. In the case of genetically‐encoded tags, the width of the distributions is considerably smaller (see also Supplementary Table 4 in the Supporting Information) due to the reduced linkage error to the actual protein location.3c, 3e, 14

**Figure 2:**
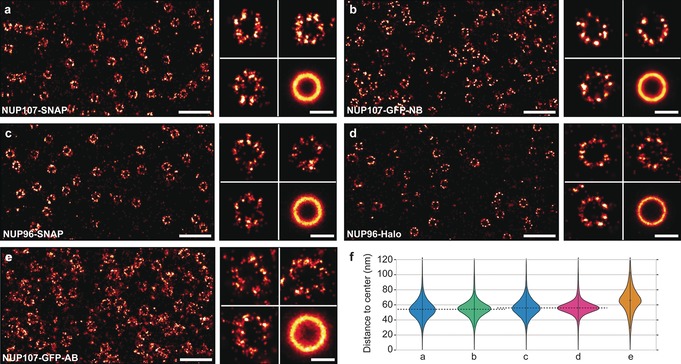
a) NUP107‐SNAP overview image (left). Zoom‐in to individual NPCs and sum image (*n*=398) (right). b) NUP107‐GFP nanobody overview image (left). Zoom‐in to individual NPCs and sum image (*n*=486) (right). c) NUP96‐SNAP overview image (left). Zoom‐in to individual NPCs and sum image (*n*=288) (right). d) NUP96‐Halo overview image (left). Zoom‐in to individual NPCs and sum image (*n*=191) (right). e) NUP107‐GFP‐Antibody overview image (left). Zoom‐in to individual NPCs and sum image (*n*=185) (right). f) Violin plots of the distances between ring center and localizations. Median radii and standard deviation were obtained for each label: NUP107‐SNAP (from **a**, 127 773 fitted localizations, 53.7±13.1 nm radius), NUP107‐GFP (from **b**, 219 398 fitted localizations, 54.6±11.9 nm radius), NUP96‐SNAP (from **c**, 57 297 fitted localizations, 55.9±12.6 nm), NUP96‐Halo (from **d**, 45 143 fitted localizations, 56.2±10.2 nm radius), NUP107‐GFP‐Anitbody (from **e**, 69 834 fitted localizations, 65.9±17.5 nm). See also Supplementary Table 2 in in the Supporting Information. Scale bars: 500 nm (overviews), 100 nm (individual NPCs and sum image).

Next, we sought out to further optimize image acquisition conditions with respect to overall localization precision, sampling of single protein sites, and three‐dimensional image acquisition (Supporting Information, Supplementary Figure 7). This allowed us to visualize single copies of NUP96 proteins (Figure 3) using the NUP96‐Halo cell line, which we chose based on its superior performance in the 2D study presented above (smallest distribution width). An overview of a typical 3D DNA‐PAINT dataset is shown in Figure 3 a. Zooming in to some of the NPCs (Figure 3 b) reveals distinctive pairs of close‐by “localization clouds” (arrows in Figure 3 b), which we attribute to single NUP96 proteins. To quantitatively asses the Euclidian distance of the two copies of NUP96 on the two cytoplasmic or nuclear rings of the NPC, we selected about 50 pairs in NPCs, aligned them on top of each other and subsequently carried out particle averaging with Picasso.2b, 15 We then performed a cross‐sectional histogram analysis of the resulting sum image and fitted the distribution with two Gaussian functions (Figure 3 c). The fit yields a peak‐to‐peak distance of about 12 nm, well in agreement with the expected distance of NUP96 proteins on adjacent Y‐complexes as derived from EM models.8a Furthermore, each peak fit exhibits a standard deviation of only circa 3 nm, highlighting the high localization precision and accuracy achievable with the combination of genetically‐encoded tags with DNA‐PAINT. Additionally, we measured the separation between the cytoplasmic and the nuclear rings for NUP96‐Halo, yielding a distance of about 61 nm (Figure 3 d), which we could clearly resolve. The capability to separate the nuclear from the cytoplasmic side of the NPC is a necessity to convince us, that the NUP pairs in each symmetry center (Figure 3 b and c) are indeed part of either the nuclear or cytoplasmic rings of the NPC. Furthermore, we obtained qualitatively and quantitatively similar results for the NUP96‐SNAP cell line (Supporting Information, Supplementary Figure 8).

**Figure 3:**
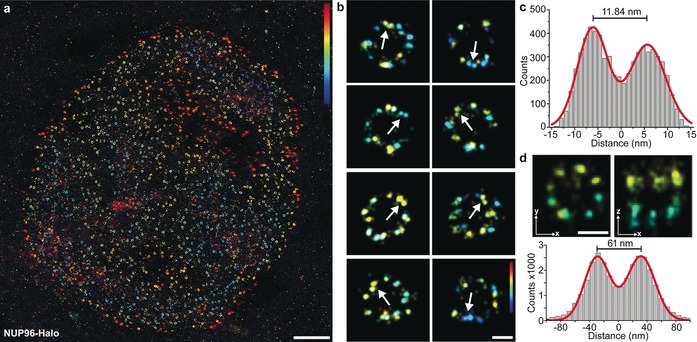
a) Overview image of NUP96‐Halo imaged using 3D DNA‐PAINT (color indicates height, range: −200 (blue) to 200 nm (red)). b) Selection of single NPCs. Arrows are highlighting two copies of NUP96 proteins in the same symmetry center of the same ring (that is at the same height) spaced ca. 12 nm apart from each other (color indicates height, range: −100 (blue) to 100 nm (red)). c) Cross‐sectional histogram of 3D‐summed pairs (*n*=45) of NUP96 proteins in single symmetry centers as highlighted by arrows in **b** yields ca.12 nm distance between single proteins. d) NUP96‐Halo‐labeled NPCs show the typical eightfold symmetry (*xy*‐projection, left) and the organization in nuclear and cytoplasmic rings (*xz*‐projection, right). Micrographs represent sum data from aligned NPCs (*n*=31). Bottom: Cross‐sectional histogram of localizations in the xz‐projection yields ca. 61 nm separation between cytoplasmic and nuclear rings. Scale bars: 2 μm (**a**), 50 nm (**b, d**).

In conclusion, we present an approach to combine DNA‐PAINT with genetically‐encoded self‐labeling tags. This provides a tool to investigate single proteins in higher order protein complexes in cells. However, we could only achieve a relatively modest labeling efficiency of approximately 30 % (Supporting Information, Supplementary Table 5). Thus, one of the main challenges in the field remains, which is a route to highly efficient labeling probes (>90 % labeling efficiency) without requiring genetic engineering. Besides the availability of peptide tags combined with nanobodies^16^ and small scaffolds like nanobodies,3a affimers,^17^ darpins,^18^ or SOMAmers^19^ novel approaches are necessary to tackle this challenge. Probes could include optimized host–guest systems,^20^ direct transient binders,^21^ or rationally‐designed small proteins.^22^ However, even with our current labeling efficiency, studies of single proteins in multicomponent complexes are within reach.

## Conflict of interest

The authors declare no conflict of interest.

## Supporting information

As a service to our authors and readers, this journal provides supporting information supplied by the authors. Such materials are peer reviewed and may be re‐organized for online delivery, but are not copy‐edited or typeset. Technical support issues arising from supporting information (other than missing files) should be addressed to the authors.

Supplementary
