# Supplementary material for: Direct Visualization of Single Nuclear Pore Complex Proteins Using Genetically‐Encoded Probes for DNA‐PAINT
Source: Angew Chem Int Ed Engl. 2019 Aug 21;58(37):13004–8. doi: 10.1002/anie.201905685 (PMC6771475; doi:10.1002/anie.201905685)
Supplement: Supplementary file 1 — Supplementary [file ANIE-58-13004-s001.pdf]

Angewandte Chemie
Eine Zeitschrift der Gesellschaft Deutscher Chemiker

Supporting Information

Direct Visualization of Single Nuclear Pore Complex Proteins Using Genetically-Encoded Probes for DNA-PAINT

*Thomas Schlichthaerle[+], Maximilian T. Strauss[+], Florian Schueder[+], Alexander Auer, Bianca Nijmeijer, Moritz Kueblbeck, Vilma Jimenez Sabinina, Jervis V. Thevathasan, Jonas Ries, Jan Ellenberg, and Ralf Jungmann**

anie_201905685_sm_miscellaneous_information.pdf

Supplementary Information

Direct visualization of single nuclear pore complex proteins using genetically-encoded probes for DNA-PAINT

**Materials and Methods**	
**Supplementary Figure 1**	**Diffraction-limited and super-resolution imaging comparison**
**Supplementary Figure 2**	**Overview of NUP107-SNAP, n=398**
**Supplementary Figure 3**	**Overview of NUP107-GFP-NB, n=486**
**Supplementary Figure 4**	**Overview of NUP96-SNAP, n=288**
**Supplementary Figure 5**	**Overview of NUP96-Halo, n=191**
**Supplementary Figure 6**	**Overview of NUP107-GFP-AB, n=200**
**Supplementary Figure 7**	**3D parameter optimization**
**Supplementary Figure 8**	**NUP96-SNAP 3D DNA-PAINT**
**Supplementary Table 1**	**Imaging Parameters**
**Supplementary Table 2**	**Imager Sequences**
**Supplementary Table 3**	**Handle Sequences**
**Supplementary Table 4**	**NPC radius quantification**
**Supplementary Table 5**	**NPC labeling efficiency**
**Supplementary References**	

Materials and Methods

Tissue culture. McCoy's 5A Medium modified (cat: 26600-023) was ordered from Gibco. Fetal Bovine Serum (FBS) (cat: 10500-064), 1× Phosphate Buffered Saline (PBS) pH 7.2 (cat: 20012-019), 0.05% Trypsin–EDTA (cat: 25300-054) and Penicillin-Streptomycin (cat: 15140-122) were purchased from Thermo Fisher Scientific. HeLa cells were purchased from the Leibniz Institute DSMZ (cat: ACC-57). U2OS cells were purchased from ATCC (cat: ATCC HTB-96). Glass-bottomed 8-well µ-slides (cat: 80827) and sticky slide VI (cat: 80608) were obtained from ibidi. Falcon tissue culture flasks (cat: 734-0965) were ordered from VWR.

Cell Fixation and immunostaining. 16% (w/v) Paraformaldehyde (cat: 28906) and DTT (cat: 20291) were purchased from Thermo Fisher Scientific. Triton X-100 (cat: 6683.1) and Ammonium chloride (cat: K298.1) was purchased from Roth. Bovine Serum Albumin (cat: A4503-10G) was ordered from Sigma-Aldrich. Halo- and SNAP-ligand-modified oligos were custom-ordered from Biomers.net (see **Supplementary Table 3**). GFP Nanobody was ordered from NanoTag Biotechnologies (cat: N0301-1mg, Clone 1H1), Secondary polyclonal antibodies (cat: 711-005-152, 115-005-003) were purchased from Jackson ImmunoResearch. Primary rabbit polyclonal anti-GFP antibody was purchased from MBL (cat: 598).

Cell imaging. EDTA 0.5 M pH 8.0 (cat: AM9261), Sodium Chloride 5 M (cat: AM9759) and Tris 1 M (cat: AM9856) were ordered from Ambion. Ultrapure water (cat: 10977-035) was purchased from Thermo Fisher Scientific. Potassium chloride (cat: 6781.1) was ordered from Roth. Sodium hydroxide (cat: 31627.290) was purchased from VWR. Glycerol (cat: G5516-500ML), Methanol (cat: 32213-2.5L), Protocatechuate 3,4-Dioxygenase Pseudomonas (PCD) (cat: P8279), 3,4-Dihydroxybenzoic acid (PCA) (cat: 37580-25G-F) and (+-)-6-Hydroxy-2,5,7,8-tetra-methylchromane-2-carboxylic acid (Trolox) (cat: 238813-5G) were purchased from Sigma-Aldrich. Dye modified DNA oligos were custom-ordered from MWG (see **Supplementary Table 4**). 90-nm-diameter Gold Nanoparticles (cat: G-90-100) were ordered from cytodiagnostics.

Cell line generation. The generation of cell lines was according to published procedures, where homozygosity of the cell lines has been verified by sequencing, Southern and Western blots. After FACS sorting and clonal expansion for all cell lines, several clones with homozygous gene replacement could be isolated after one round of CRISPR facilitated recombination. The NUP96 cell line was furthermore described elsewhere[1].

Buffers. The imaging buffer was supplemented with: 100× Trolox: 100 mg Trolox, 430 µl 100 % Methanol, 345 µl 1M NaOH in 3.2 ml H_2O. 40× PCA: 154 mg PCA, 10 ml water and NaOH were mixed and pH was adjusted 9.0. 100× PCD: 9.3 mg PCD, 13.3 ml of buffer (100 mM Tris-HCl pH 8, 50 mM KCl, 1 mM EDTA, 50 % Glycerol). Cell-imaging-buffer (buffer C): 1× PBS pH 7.2, 500 mM NaCl, 1× PCA, 1× PCD, 1× Trolox.

PEG surface. PEG surfaces were prepared as previously reported[2]. In brief, the coverslips (no. 1.5 high precision, 60x18 mm^2) were rinsed twice and bath-sonicated in a Teflon-based custom-made slide holder in Milli-Q water for 10 min. Rinsing and bath-sonication was repeated with methanol and acetone. To activate the surface, the coverslips were bath-sonicated in 1 M KOH for 20 min and rinsed with Milli-Q water afterwards. After blow drying the coverslips with nitrogen, they were incubated with 95 ml methanol mixed with 5 ml acetic acid and 1 ml aminosilane for 20 min in the dark. Afterwards they were washed with methanol and water for 2 min and blow dried with nitrogen. The aminosilanized coverslips were stored under Argon atmosphere for <2 weeks until they were used. 24 h before use, the imaging chambers were assembled with double sided sticky tape. 16 mg of mPEG (Rapp Polymere, cat. no.: 125000-35) was dissolved in 70 µl of freshly prepared sodium bicarbonate buffer (10 mM sodium bicarbonate, pH 8.5) and mixed 20:1 with biotin-PEG (Rapp Polymere, cat. no.: 135000-25-35). To remove air bubbles, the mix was briefly spun down for 30 s and added to the assembled chamber. The chamber was sealed with silicon (picodent) and stored at room temperature overnight in the dark. Before use, the chamber was washed with 1 ml Milli-Q water.

Cysteine-based GFP-Nanobody labeling and purification. GFP nanobodies were DNA-labeled as previously reported[3]. Nanobodies were concentrated via Amicon 10 kDa spin filters and buffer exchanged into 5 mM TCEP in 1× PBS + 3 mM EDTA at pH 7.5. 5 mM TCEP in 1× PBS + 3 mM EDTA was then added to the GFP Nanobody and was incubated for 2 h at 4 °C on a shaker. Subsequently, Amicon 10 kDa Spin Filters were prewashed with 1× PBS, and Nanobody was buffer-exchanged into 1× PBS for 5× 5 min at 14 000×g and the volume was adjusted to 100 µl. DBCO-Maleimide Crosslinker was added in 20 molar excess in 5 µl to the GFP Nanobody and incubated overnight at 4 °C on a shaker. Crosslinker aliquots were stored at 40 mg/ml concentration in DMF. DBCO crosslinker was removed via 10 kDa Amicon Spin Filters for 5× 5min at 14 000× g. Azide-DNA was added to the GFP Nanobody crosslinker at 10× excess for 1 h at 20 °C. The final product was buffer exchanged into Anion exchange binding buffer (1× PBS, pH 7.2) via Amicon 10 kDa Spin Filters. Purification from free DNA was performed using a GE Aekta purifier system and a RESOURCE Q 1 ml column via a 30 min gradient purification scheme from 1× PBS to 1× PBS + 1 M NaCl. Peak fractions were afterwards concentrated and buffer-exchanged via Amicon 10 kDa spin filters into 1× PBS.

Antibody conjugation. Antibodies were conjugated to DNA-PAINT docking sites via maleimide-PEG2-succinimidyl ester chemistry as previously reported[4] (see **Supplementary Table 3** for handle sequences). In short, secondary antibodies were concentrated via 100 kDa amicon spin filters to a final concentration of 1-3 mg/ml. 100 µl of antibody was labelled with the maleimide-Peg2-succinimidyl ester for 90 min at 10x molar excess at 4 °C on a shaker. Crosslinker stocks of 10 mg/ml in DMF were diluted in 1x PBS to reach 10x molar excess in 5 µl, which were subsequently added to the antibody. After the reaction had been done, unreacted crosslinker was removed via a zeba spin column. Thiolated DNA was reduced using DTT for 2 h at room temperature. DTT was purified from the reduced DNA via a Nap5 column and fractions containing DNA were concentrated via 3 kDa amicon spin filters. The reduced DNA was then added to the antibody bearing a functional maleimide group in 10x molar excess and incubated over night at 4 °C on a shaker in the dark. Antibody-DNA constructs were finally purified via 100 kDa amicon.

Cell culture. Hela cells and U2OS cells were passaged every other day and used between passage number 5 and 20. The cells were maintained in DMEM supplemented with 10 % Fetal Bovine Serum and 1 % Penicillin/Streptomycin. Passaging was performed using 1× PBS and Trypsin-EDTA 0.05 %. 24 h before immunostaining, cells were seeded on ibidi 8-well glass coverslips at 30,000 cells/well.

Cell fixation. Prefixation was performed with prewarmed 2.4 % Paraformaldehyde for 20 seconds followed by the permeabilization at 0.4 % Trion-X 100 for 10 seconds. Next, cells were fixed (main fixation) with 2.4 % for 30 min. After 3× rinsing with 1× PBS the cells were quenched with 50 mM Ammoniumchloride (in 1× PBS) for 4 minutes. Then, cells were washed 3× with 1×PBS followed by incubation in 1× PBS for 5 minutes twice. Next, cells were stained with the corresponding ligand (see below). Finally, cells were washed 3× for 5 min in 1× PBS, incubated with 1:1 dilution of 90 nm gold particles in 1× PBS as drift markers, washed 3× 5 min and immediately imaged.

Staining with SNAP. For SNAP-labeling, cells were incubated with 1 µM of SNAP-ligand-modified DNA oligomer in 0.5 % BSA and 1 mM DTT for 2 hours.

Staining with Halo. For Halo-labeling, cells were incubated with 1 µM Halo-ligand-modified DNA oligomer in 3 % (w/v) BSA in 1× PBS for overnight at 4°C on a shaker.

Staining with GFP. GFP-Nanobody staining was done in 3% BSA in 1xPBS at 4°C overnight on a shaker.

Staining with antibodies. Antibody staining was done in two steps. First, cells were incubated with primary antibody anti-GFP (1:100) in 3% BSA at 4°C PBS overnight. After three washes for 5 min with 1× PBS, the sample was incubated with the secondary antibody (dilution 1:100) at RT for 1 hours.

Super-resolution microscope setup. Fluorescence imaging was carried out on an inverted Nikon Eclipse Ti microscope (Nikon Instruments) with the Perfect Focus System, applying an objective-type TIRF configuration with an oil-immersion objective (Apo SR TIRF 100×, NA 1.49, Oil). TIRF/Hilo angle was adjusted for highest signal to noise ratio when imaging. A 561 nm (200 mW, Coherent Sapphire) laser was used for excitation. The laser beam was passed through cleanup filters (ZET561/10, Chroma Technology) and coupled into the microscope objective using a beam splitter (ZT561rdc, Chroma Technology). Fluorescence light was spectrally filtered with an emission filter (ET600/50m and ET575lp, Chroma Technology) and imaged on a sCMOS camera (Andor Zyla 4.2) without further magnification, resulting in an effective pixel size of 130 nm (sCMOS after 2×2 binning).

Imaging conditions

Figure 1c-e. Imaging was carried out using an imager strand concentration of 300 pM (P3-Cy3B) in cell imaging buffer. 15,000 frames were acquired at 200 ms integration time. The readout bandwidth was set to 200 MHz. Laser power (@561 nm) was set to 30 mW (measured before the back focal plane (BFP) of the objective), corresponding to 0.7 kW/cm^2 at the sample plane.

Figure 1f-h. Images were acquired with an imager strand concentration of 2 nM (P3-Cy3B imager) in cell imaging buffer. 40,000 frames were acquired at 200 ms integration time. The readout bandwidth was set to 200 MHz. Laser power (@561 nm) was set to 80 mW (measured at the back focal plane (BFP) of the objective), corresponding to 1.8 kW/cm^2 at the sample plane.

Figure 2a. Images were acquired with an imager strand concentration of 2 nM of P3-Cy3B in cell imaging buffer. 30,000 frames were acquired at 200 ms integration time and a readout bandwidth of 200 MHz. Laser power (@560 nm) was set to 50 mW (measured before the back focal plane (BFP) of the objective), corresponding to 1.1 kW/cm^2 at the sample plane.

Figure 2b. Imaging was carried out using an imager strand concentration of 2 nM (P3-Cy3B) in cell imaging buffer. 30,000 frames were acquired at 200 ms integration time. The readout bandwidth was set to 200 MHz. Laser power (@561 nm) was set to 50 mW (measured before the back focal plane (BFP) of the objective), corresponding to 1.1 kW/cm^2 at the sample plane.

Figure 2c. Images were acquired with an imager strand concentration of 2 nM (P3-Cy3B imager) in cell imaging buffer. 30,000 frames were acquired at 200 ms integration time. The readout bandwidth was set to 200 MHz. Laser power (@561 nm) was set to 50 mW (measured at the back focal plane (BFP) of the objective), corresponding to 1.1 kW/cm^2 at the sample plane.

Figure 2d. Images were acquired with an imager strand concentration of 2 nM of P3-Cy3B in cell imaging buffer. 30,000 frames were acquired at 200 ms integration time. The readout bandwidth was set to 200 MHz. Laser power (@561 nm) was set to 50 mW (measured at the back focal plane (BFP) of the objective), corresponding to 1.1 kW/cm^2 at the sample plane.

Figure 2e. Imaging was carried out using an imager strand concentration of 300 pM (P3-Cy3B) in cell imaging buffer. 30,000 frames were acquired at 200 ms integration time. The readout bandwidth was set to 200 MHz. Laser power (@561 nm) was set to 50 mW (measured before the back focal plane (BFP) of the objective), corresponding to 1.1 kW/cm^2 at the sample plane.

Figure 3a, b. Images were acquired with an imager strand concentration of 2 nM (P3-Cy3B imager) in cell imaging buffer. 100,000 frames were acquired at 200 ms integration time. The readout bandwidth was set to 200 MHz. Laser power (@561 nm) was set to 40 mW (measured at the back focal plane (BFP) of the objective), corresponding to 1.0 kW/cm^2 at the sample plane.

For all imager strand sequences see **Supplementary Table 4**.

3D DNA-PAINT calibration using latex microspheres. The 3D look-up table was measured as previously reported[5]. In short, first an ibidi sticky slide VI was assembled with the pegylated coverslip. Then, 50 µl of 1:10 avidin coated microspheres diluted in 1× PBS were flown into the ibidi sticky slide chamber with the prepared PEG-Biotin surface and incubated for 10 min. Then the chamber was washed using 180 µl of 1x PBS. Second, 500 nM biotinylated oligonucleotides (10 nt, P1 docking site sequence, **Supplementary Table 4**) was then flown into the chamber and incubated for 10 min. Next, the chamber was washed with 180 µl of 1× PBS. Next, the chamber was incubated with 1:10 dilution of 90 nm gold particles in 1× PBS as drift markers for 5 min and subsequently washed with 80 µl 1× PBS. Finally, 180 µl imaging buffer with dye-labeled imager strands was flown into the chamber. 500 pM Cy3B labeled imager with sequence P1 and 1× PCA, 1× PCD, 1× Trolox in buffer C was used. Latex microspheres attached to the PEG surface were identified using bright-field illumination and the radius was measured. The recoded latex microsphere data using DNA-PAINT was reconstructed using two-dimensional gaussian fitting. Lateral drift correction was performed using the gold nanoparticle. Gaussian width (sigma x and sigma y) were averaged in radial sections and linked to the corresponding z height to gather the calibration data[6]. Finally, the calibration data was fitted using sixth degree polynomial fit to generate the look-up table.

Image analysis. Raw fluorescence data was subjected to spot-finding and subsequent super-resolution reconstruction using the ‘Picasso’ software package[4b]. x, y drift correction was performed via a redundant cross-correlation and gold particles as fiducial markers. Drift correction in z was performed via 90 nm gold particles.

Radius analysis. To determine the radius of NPCs, picked NPCs were averaged using the ‘Picasso:average3’ module as previously described[4b]. In brief, localizations of particles are aligned on top of each other by rendering them and using cross-correlation to determine displacement. To account for ring-like structures, a 100× symmetry was set. Each dataset was averaged with the following oversampling settings: 3× 15, 1× 20, 1× 40. Based on the resulting “superparticle”, the center of mass was determined. The localizations were subsequently transformed into polar coordinates with the center of mass as the center point. The radius was calculated by taking the median of the polar coordinate distances.

Labeling efficiency calculation. To analyse the labelling efficiency for the different labelling methods (NUP96-Halo, NUP96-SNAP, NUP107-SNAP, NUP107-GFP). 100 nuclear pores were picked for each 3D dataset, and the apparent clusters were counted and compared to the expected 32 copies of the proteins.

Parameter optimization for 3D imaging. For parameter optimization two main aspects for DNA-PAINT imaging should be considered: Resolution (x, y, z) and repetitive sampling. To estimate the resolution capabilities, 125 single clusters in nuclear pores were picked and center-of-mass-aligned with the Picasso average3 module. Localization distributions of aligned clusters were fitted with a Gaussian fit. For the analysis of repetitive sampling, the picked single 125 clusters were linked (max. distance: 26 nm, max. transient dark frames: 5) and DBSCANned (minimum local event density: 5, Radius: 26 nm) using the Picasso software to obtain the events per site as well as mean frame of the visits.

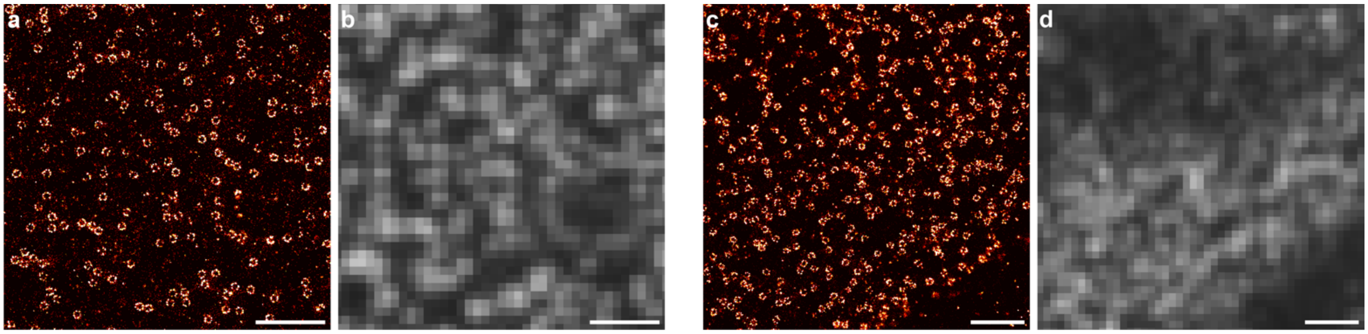

Supplementary Figure 1 | Diffraction-limited and super-resolution imaging comparison. (**a**) NUP96-SNAP super-resolved image. (**b**) Diffraction-limited image of the same area. (**c**) NUP107-GFP-NB super-resolved image. (**d**) Diffraction-limited image of the same area. Scale bars: 1 µm.

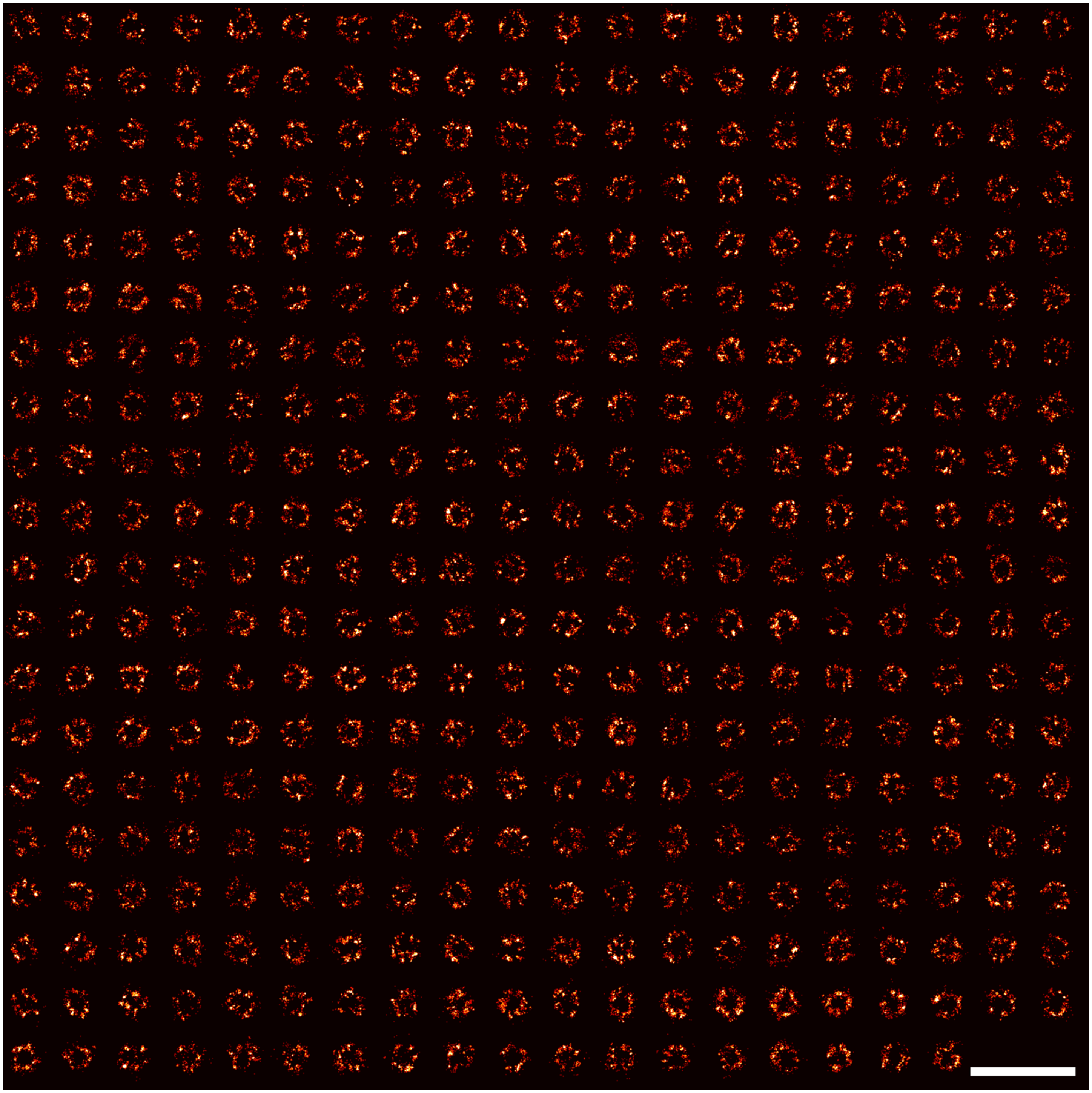

Supplementary Figure 2 | Overview of NUP107-SNAP, n=398. Scale bar: 500 nm.

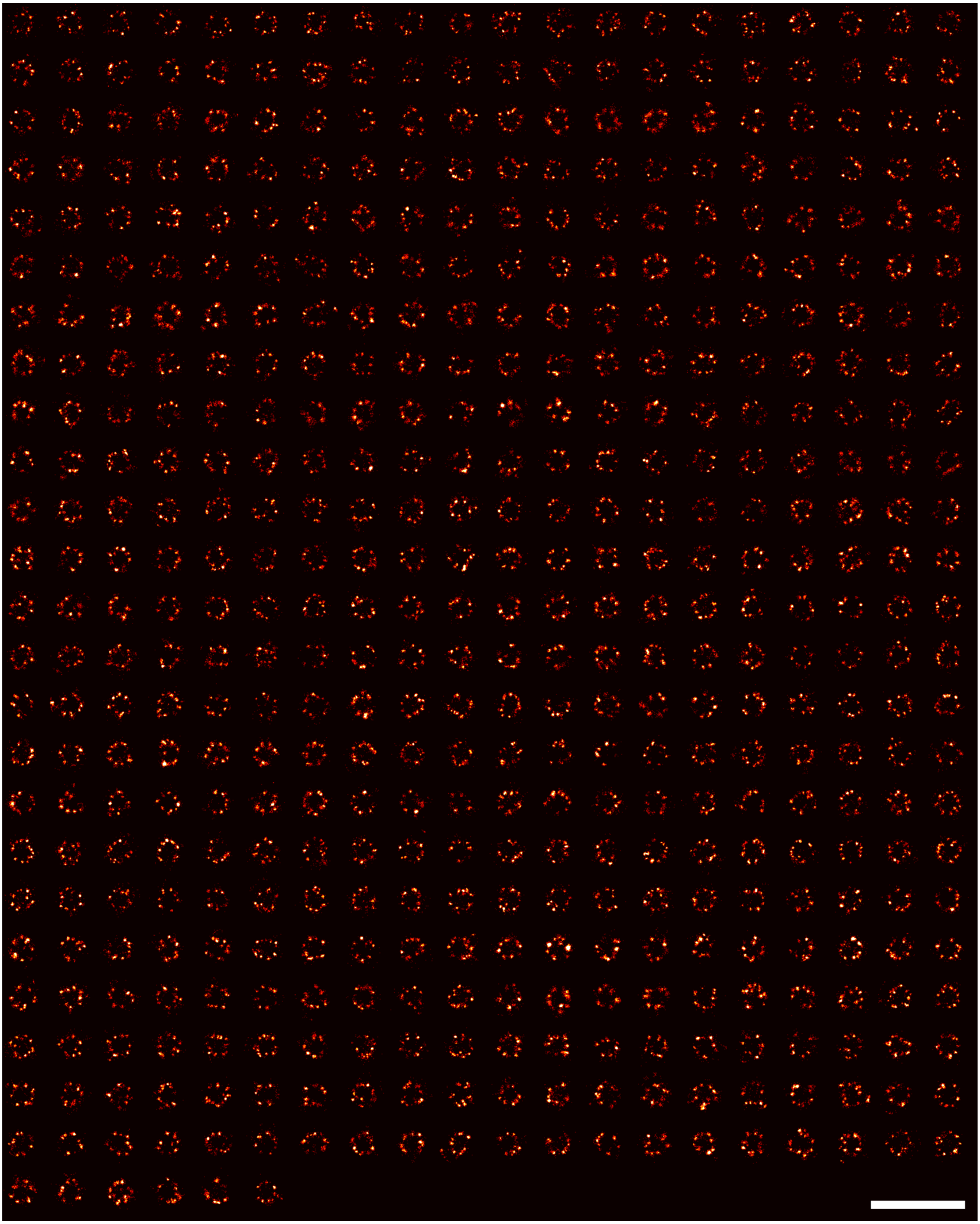

Supplementary Figure 3 | Overview of NUP107-GFP-NB, n=486. Scale bar: 500 nm.

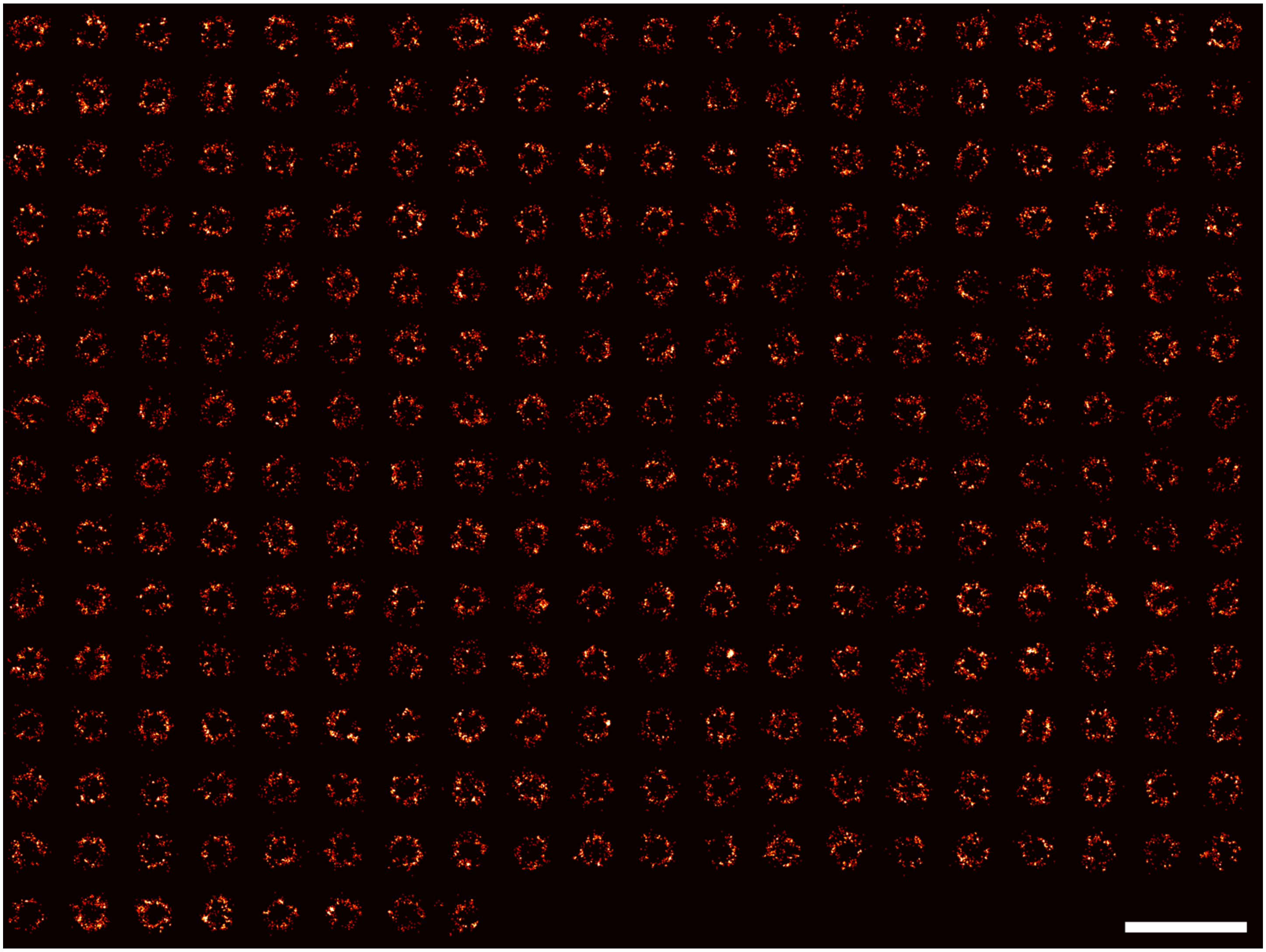

Supplementary Figure 4 | Overview of NUP96-SNAP, n=288. Scale bar: 500 nm.

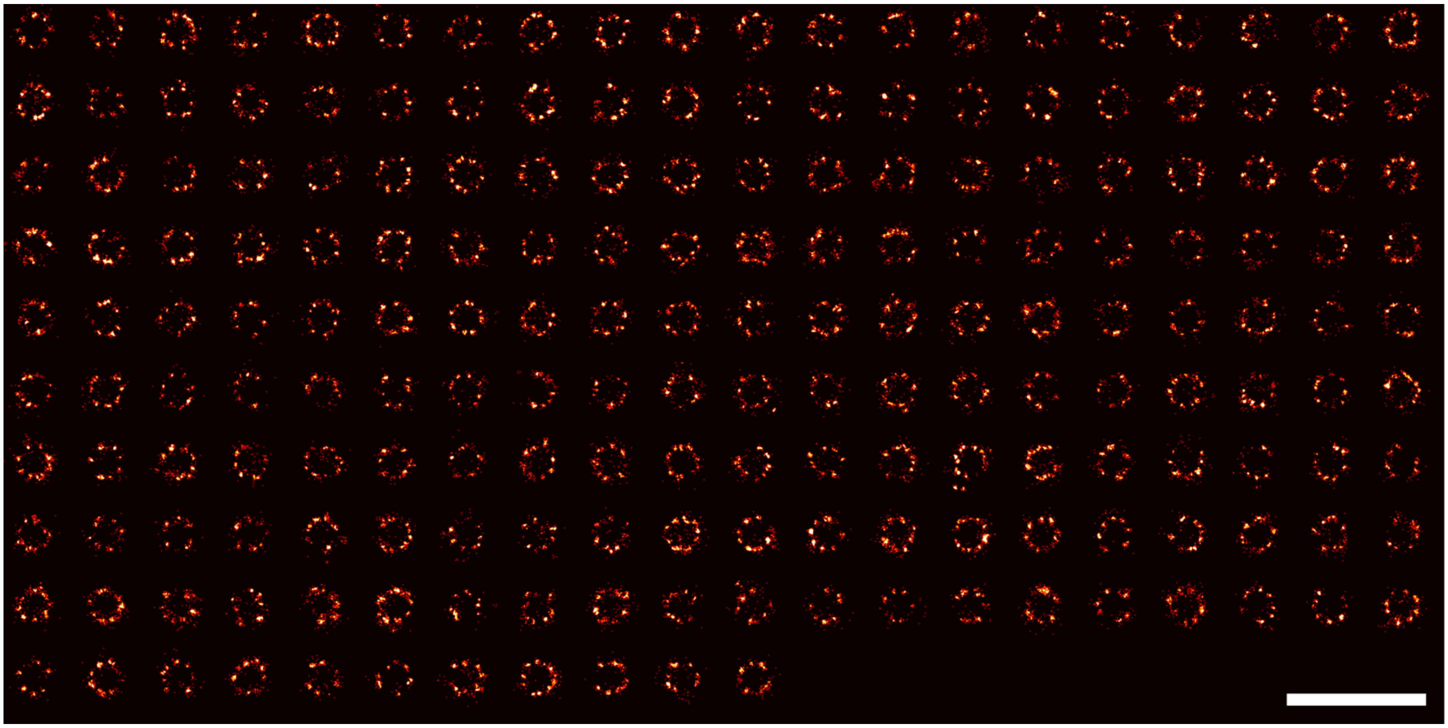

Supplementary Figure 5 | Overview of NUP96-Halo, n=191. Scale bar: 500 nm.

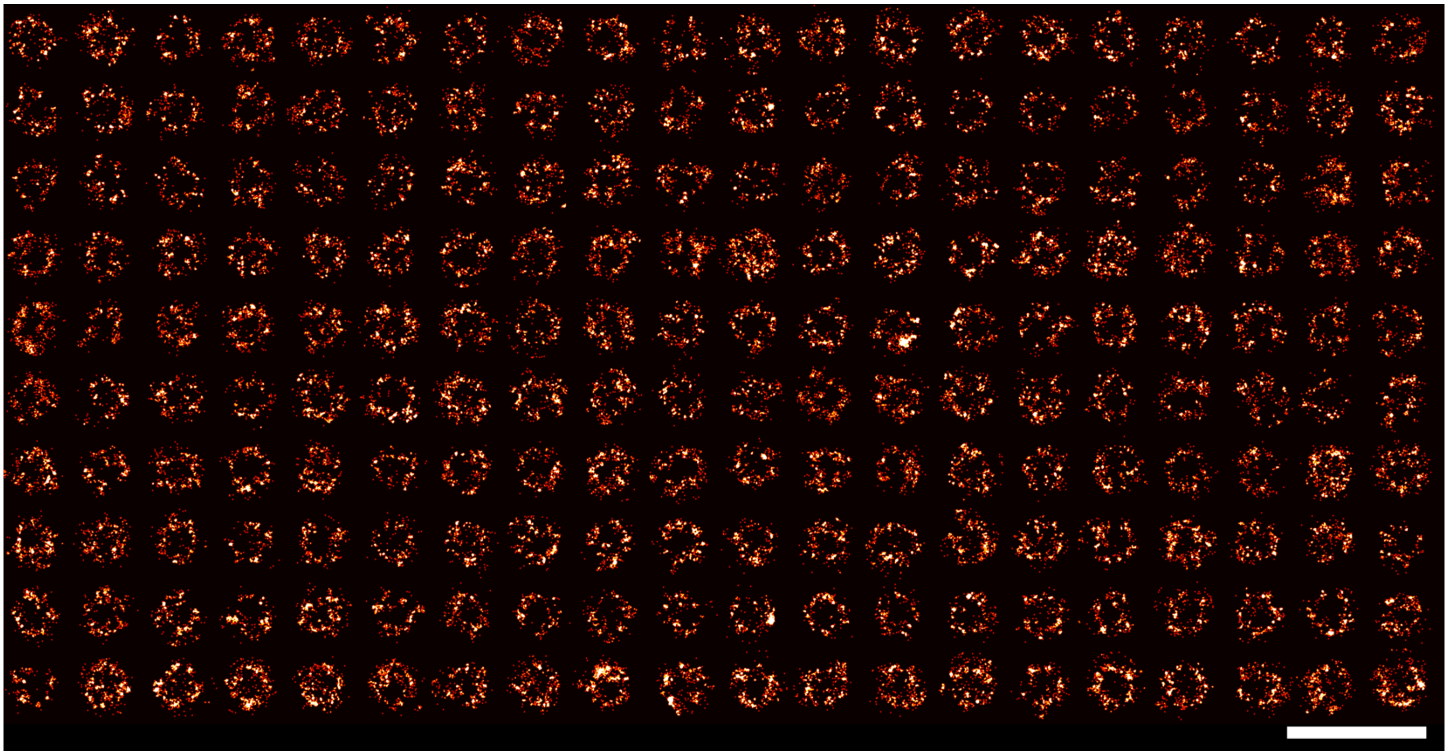

Supplementary Figure 6 | Overview of NUP107-GFP-AB, n=200. Scale bar: 500 nm.

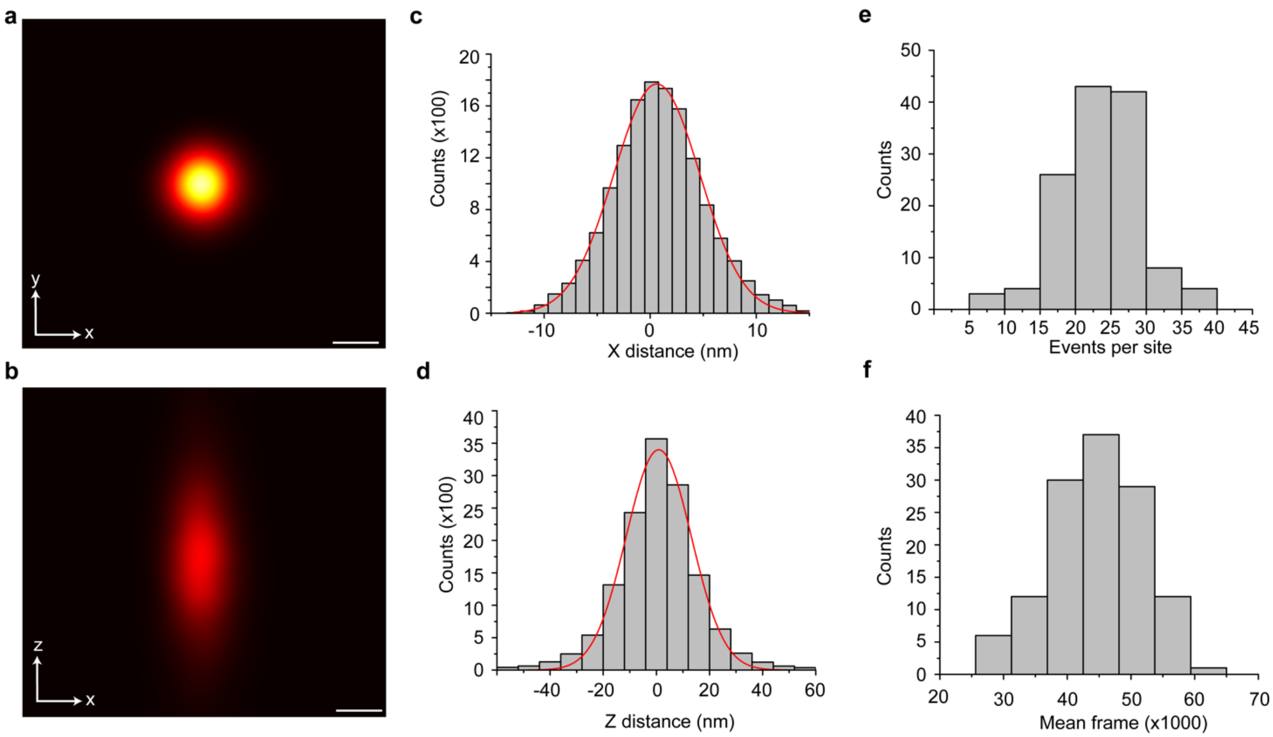

Supplementary Figure 7 | 3D parameter optimization. For optimization of imaging parameters to resolve the two copies of NUP96 proteins spaced ~12 nm apart, certain criteria need to be fulfilled, which are high spatial resolution as well as sufficient repetitive sampling of binding sites. (**a**) Center of mass alignment of 125 single protein clusters (x-y projection). (**b**) Center-of-mass alignment of the single protein clusters (x-z projection). (**c**) Histogram analysis of the x-y projection yields ~4 nm localization precision. (**d**) Histogram analysis of the x-z projection yields ~12 nm localization precision. (**e**) Histogram of events per cluster yields a mean of 23.0 (STD: 5.7) visits of imager strands per site. (**f**) Histogram analysis of the mean frame per cluster yields a mean value of 44008 (STD: 8627), highlighting that the clusters were repetitively visited over the whole course of image acquisition (100k frames). Analysis based on Figure 3 dataset. Scale bars: 10 nm (**a, b**).

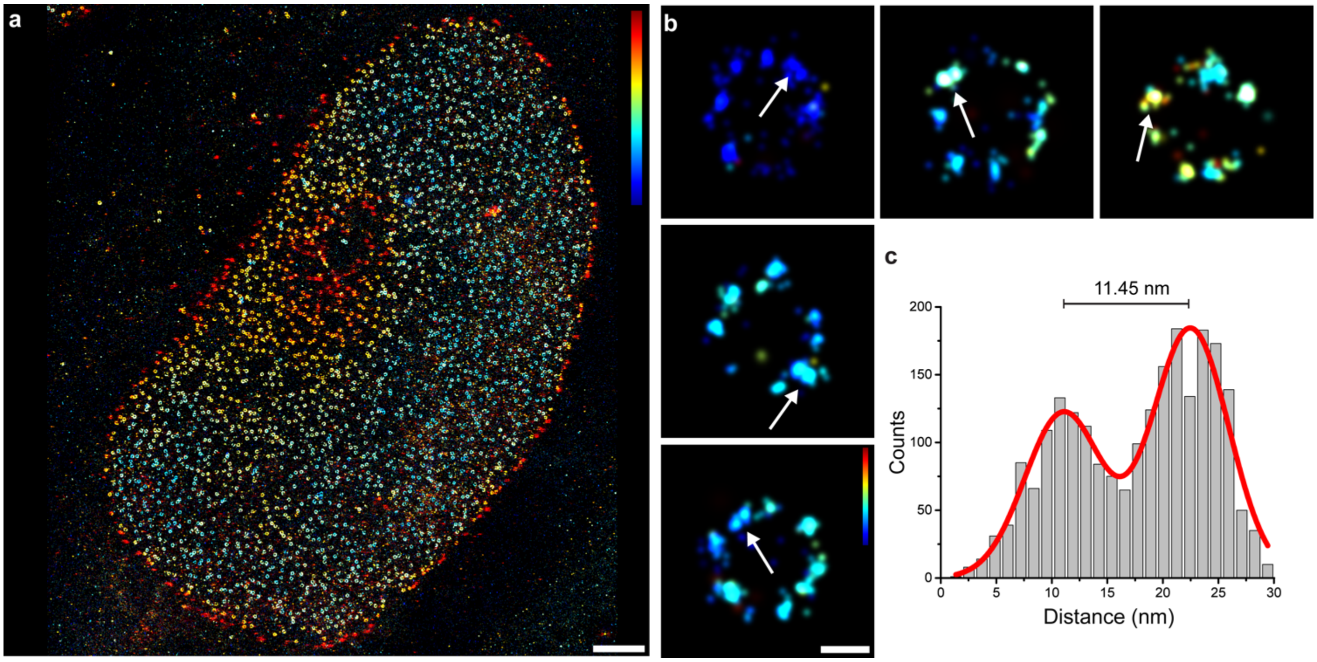

Supplementary Figure 8 | NUP96-SNAP 3D DNA-PAINT. (**a**) 3D DNA-PAINT overview image of NPCs labeled via Nup96-SNAP (color indicates height, range: -200 (blue) to 200 nm (red)). (**b**) Selection of single NPCs. Arrows are highlighting two copies of NUP96 proteins in the same symmetry center of the same ring (i.e. at the same height) spaced ~12 nm apart from each other (color indicates height, range: -100 (blue) to 100 nm (red)). (**c**) Cross sectional histogram of 3D-averaged pairs (N = 27) of NUP96-SNAP proteins in single symmetry centers as highlighted in (**b**). Scale bars: 2 µm (**a**), 50 nm (**b**).

Imaging was carried out using an imager strand concentration of 2 nM (P3-Cy3B). 50k frames were acquired at 200 ms integration time. The readout bandwidth was set to 200 MHz. Laser power (@561 nm) was set to 40 mW (measured before the back focal plane (BFP) of the objective). This corresponds to 1 kW/cm^2 at the sample plane.

Supplementary Table 1 | Imaging parameters

Dataset	Parameters	Power @561 nm	NeNA precision
Figure 1c,d and SI Figure 1a,b	300ms, 2D, 15k Frames, 5nM. P3*	0.7 kW/cm^2	6.3 nm
Figure 1e,f and SI Figure 1c,d	200ms, 2D, 30k Frames, 2nM, P3*	1.8 kW/cm^2	6.9 nm
Figure 2a and SI Figure 2	200 ms, 2D, 30k Frames, 1 nM, P3*	1.1 kW/cm^2	3.6 nm
Figure 2b and SI Figure 3	200 ms, 2D, 30k Frames, 2nM, P3*	0.6 kW/cm^2	6.0 nm
Figure 2c SI Figure 4	200 ms, 2D, 30k Frames, 1 nM, P3*	1.1 kW/cm^2	2.9 nm
Figure 2d and SI Figure 5	200 ms, 2D, 30k Frames, 1 nM, P3*	1.1 kW/cm^2	4.5 nm
Figure 2e and SI Figure 6	200ms, 2D, 30k Frames, 300 pM, P3*	0.6 kW/cm^2	4.5 nm
Figure 3 and SI Figure 7	200ms, 3D, 100k Frames, 2nM, P3*	1 kW/cm^2	6.0 nm
SI Figure 6a	200 ms, 2D, 30k Frames, 300 pM, P3*	1.1 kW/cm^2	2.2 nm
SI Figure 6b	200 ms, 2D, 30k Frames, 1 nM, P3*	1.1 kW/cm^2	6.0 nm
SI Figure 8	200 ms, 3D, 50k Frames, 2 nM, P3*	1 kW/cm^2	5.1 nm

Supplementary Table 2 | Imager sequences

Imager name	Sequence	5'-mod	3'-mod	Vendor
P1*	CTAGATGTAT	None	Cy3b	Eurofins Genomics
P3*	GTAATGAAGA	None	Cy3b	Eurofins Genomics

Supplementary Table 3 | Handle sequences

Handle Name	Sequence	5'-mod	3'-mod	Vendor
P1	TTATACATCTA	BG (Snap Ligand)	None	Biomers.net
P3	TTTCTTCATTA	BG (Snap Ligand)	None	Biomers.net
P1	TTATACATCTA	Halo Ligand (O2)	None	Biomers.net
P3	TTTCTTCATTA	Halo Ligand (O2)	None	Biomers.net
P1	TTATACATCTA	Thiol (for AB conjugation)	None	Eurofins Genomics
P3	TTTCTTCATTA	Thiol (for AB conjugation)	None	Eurofins Genomics
P1	TTATACATCTA	Biotin	None	Eurofins Genomics

Supplementary Table 4 | NPC radius quantification, SE = standard error

Dataset	Median (nm)	Mean (nm)	Std (nm)	SE of Median (nm)	SE of Mean (nm)	# Pores	# Locs
NUP107-SNAP	53.7	54.2	13.1	0.8	0.7	398	127773
NUP107-GFP-NB	54.6	54.8	11.9	0.7	0.5	486	219398
NUP96-SNAP	55.9	56.5	12.6	0.9	0.7	288	57297
NUP96-Halo	56.2	56.6	10.2	0.9	0.7	191	45143
NUP107-GFP-AB	65.9	66.1	17.5	1.5	1.2	200	69834

Supplementary Table 5 | NPC labeling efficiency (LE) estimation

Dataset	Mean LE (%)	Median LE (%)	Std (%)	# Pores
NUP107-SNAP	28.1	28.1	4.9	100
NUP107-GFP-NB	27.5	28.1	4.4	100
NUP96-SNAP	28.2	28.1	4.1	100
NUP96-Halo	29.7	31.3	5.4	100

Supplementary References

[1] J. V. Thevathasan, M. Kahnwald, K. Cieśliński, P. Hoess, S. K. Peneti, M. Reitberger, D. Heid, K. C. Kasuba, S. J. Hoerner, Y. Li, Y.-L. Wu, M. Mund, U. Matti, P. M. Pereira, R. Henriques, B. Nijmeijer, M. Kueblbeck, V. J. Sabinina, J. Ellenberg, J. Ries, *bioRxiv* **2019**, 582668.

[2] A. Jain, R. Liu, Y. K. Xiang, T. Ha, *Nat Protoc* **2012**, *7*, 445-452.

[3] T. Schlichthaerle, A. S. Eklund, F. Schueder, M. T. Strauss, C. Tiede, A. Curd, J. Ries, M. Peckham, D. C. Tomlinson, R. Jungmann, *Angew Chem Int Ed Engl* **2018**, *57*, 11060-11063.

[4] aS. S. Agasti, Y. Wang, F. Schueder, A. Sukumar, R. Jungmann, P. Yin, *Chem Sci* **2017**, *8*, 3080-3091; bJ. Schnitzbauer, M. T. Strauss, T. Schlichthaerle, F. Schueder, R. Jungmann, *Nat Protoc* **2017**, *12*, 1198-1228.

[5] A. Auer, T. Schlichthaerle, J. B. Woehrstein, F. Schueder, M. T. Strauss, H. Grabmayr, R. Jungmann, *Chemphyschem* **2018**, *19*, 3024-3034.

[6] C. Cabriel, N. Bourg, G. Dupuis, S. Leveque-Fort, *Opt Lett* **2018**, *43*, 174-177.
